# IL-15 induced bystander activation of CD8^+^ T cells may mediate endothelium injury through NKG2D in Hantaan virus infection

**DOI:** 10.3389/fcimb.2022.1084841

**Published:** 2022-12-15

**Authors:** Xiyue Zhang, Yusi Zhang, He Liu, Kang Tang, Chunmei Zhang, Meng Wang, Manling Xue, Xiaozhou Jia, Haifeng Hu, Na Li, Ran Zhuang, Boquan Jin, Fanglin Zhang, Yun Zhang, Ying Ma

**Affiliations:** ^1^ Department of Immunology, Basic Medicine School, Air-Force Medical University (The Fourth Military Medical University), Xi’an, China; ^2^ Basic Medicine School, Yanan University, Yan’an, China; ^3^ Department of Microbiology, Basic Medicine School, Air-Force Medical University (The Fourth Military Medical University), Xi’an, China; ^4^ Department of Infectious Diseases, Eighth Hospital of Xi'an, Xi’an, China; ^5^ Center for Infectious Diseases, Tangdu Hospital, Air-Force Medical University (The Fourth Military Medical University), Xi’an, China; ^6^ Department of Transfusion Medicine, Xijing Hospital, Air-Force Medical University (The Fourth Military Medical University), Xi’an, China

**Keywords:** CD8^+^ T cell, bystander activation, IL-15, NKG2D, HTNV, hemorrhagic fever with renal syndrome, endothelial cell

## Abstract

**Introduction:**

Hantaan virus (HTNV) can cause endothelium injury in hemorrhagic fever with renal syndrome (HFRS) patients. Bystander activation of CD8+ T cells by virus infection has been shown that was involved in host injury, but it is unclear during HTNV infection. This project aimed to study the effect of bystander-activated CD8+ T cell responses in HTNV infection.

**Methods:**

The in vitro infection model was established to imitate the injury of endothelium in HFRS patients. Flow cytometry was performed to detect the expression of markers of tetramer+ CD8+ T cells and human umbilical vein endothelial cells (HUVECs). The levels of interleukin-15 (IL-15) in serum and supermanant were detected using ELISA kit. The expression of MICA of HUVECs was respectively determined by flow cytometry and western blot. The cytotoxicity of CD8+ T cells was assessed through the cytotoxicity assay and antibody blocking assay.

**Results:**

EBV or CMV-specific CD8+ T cells were bystander activated after HTNV infection in HFRS patients. HTNV-infected HUVECs in vitro could produce high levels of IL-15, which was positively correlated with disease severity and the expression of NKG2D on bystander-activated CD8+ T cells. Moreover, the elevated IL-15 could induce activation of CD122 (IL-15Rβ)+NKG2D+ EBV/CMV-specific CD8+ T cells. The expression of IL-15Rα and ligand for NKG2D were upregulated on HTNV-infected HUVECs. Bystander-activated CD8+ T cells could exert cytotoxicity effects against HTNV-infected HUVECs, which could be enhanced by IL-15 stimulation and blocked by NKG2D antibody.

**Discussion:**

IL-15 induced bystander activation of CD8+ T cells through NKG2D, which may mediate endothelium injury during HTNV infection in HFRS patients.

## Introduction

Hantavirus, belonging to the family Bunyaviridae, cause severe hemorrhagic fever with renal syndrome (HFRS) or hantavirus cardiopulmonary syndrome (HCPS) in humans (Kim et al., 2020; Kell, 2022). The Old World hantaviruses, including Puumala virus, Hantaan virus (HTNV), Seoul virus, and Dobrava virus are the major causative agents of HFRS in Europe and Asia (Avsic-Zupanc et al., 2019). While the New World hantaviruses, such as Andes virus and Sin Nombre virus always cause HCPS cases in America (Ma et al., 2020; Mir, 2021). HTNV is the etiological agent of HFRS in China (Tariq and Kim, 2022). Annually, 150,000–200,000 clinical cases of hantavirus infection are reported worldwide with mortality rates of HFRS and HCPS up to 15% and 50%, respectively (Mir, 2021). According to the report of the Chinese Center for Disease Control and Prevention, a total of 106,939 HFRS cases were reported during 2012–2021 in China (Statistics, 2021; Yang et al., 2022), contributing to more than 80% of the global cases. Therefore, studies on the immune response in HTNV infection could help us to better understand the pathogenesis of HFRS and develop better treatment for HFRS patients.

Hantaviruses are enveloped negative-sense single-stranded RNA viruses. Hantavirus primarily infects human vascular endothelial cells (ECs) but does not cause direct cytopathic effects on infected cells (Tariq and Kim, 2022). The leakage of vascular ECs could lead to the increased permeability of capillaries and small vessels, which were associated with clinical symptoms of HFRS such as hypotension and acute kidney injury (Avsic-Zupanc et al., 2019). It has been widely considered that the immune responses, especially T cells’ response after HTNV infection may be closely correlated with the pathogenesis of HFRS (Tariq and Kim, 2022). Our previous studies found that HTNV-specific CD8^+^ T cells could provide protective immunity for viral clearance both in HFRS patients and in animal infection models (Ma et al., 2016; Tang et al., 2017; Ma et al., 2020). However, Kenta et al. revealed that the HTNV strain Korean hemorrhagic fever virus could cause HFRS symptoms such as renal hemorrhagic in wide-type mice. But the same symptoms were not observed in CD8^+^ T cell-depleted mice (Shimizu et al., 2018). In addition, the other study showed that the increased vascular permeability of HFRS patients may be caused by the pathological responses of activated CD8^+^ T cells during HTNV infection (Tariq and Kim, 2022). According to the above, there are two-sides effects of CD8^+^ T cell responses during HTNV infection. However, the mechanisms of activated CD8^+^ T cell responses for protection or for immunopathological injury during HTNV infections were still unknown.

Bystander activation of CD8^+^ T cells has been reported in many microbial infections both in human and animal models (Sandalova et al., 2010; Odumade et al., 2012; Younes et al., 2016; Kim et al., 2018). The pre-existing memory CD8^+^ T cells that are non-specific to the infectious pathogens could be activated in a cytokine-dependent manner without T cell receptor (TCR) stimulation, which may mediate host injury during the early stages of viral infections (Lee et al., 2022). It was found that the activation and proliferation of bystander CD8^+^ T cells can be induced by interleukin-15 (IL-15) (Nolz and Richer, 2020), which was produced by the viral-infected cells during hepatitis A virus (HAV) infection (Kim et al., 2018). The IL-15 bind with IL-15 α-type receptor (IL-15Rα) on cells such as monocytes and viral-infected cells, and trans-present to NK cells or memory CD8^+^ T cells which expressed the IL-15 β-type receptor (IL-15Rβ) (CD122)–γ (CD132) chain heterodimeric receptors (Yang and Lundqvist, 2020). Notably, IL-15 could enhance NK cell activating receptors-dependent cytotoxicity of bystander-activated CD8^+^ T cells, which was referred to as “innate-like (or NK-like) cytotoxicity” (Correia et al., 2011).

Natural-killer group 2, member D (NKG2D, CD314) is one of the main NK cell-activating receptors responsible for the innate-like cytotoxicity of bystander-activated CD8^+^ T cells (Suarez et al., 2020). Studies have revealed that IL-15 could induce the expression of NKG2D on CD8^+^ T cells in a strong dose-dependent manner in the absence of TCR engagement, resulting in the differentiation from naive CD8^+^ T cell to NKG2D^high^CD8^+^ T cell (Ruck et al., 2015; Vuletic et al., 2020). Indeed, it has been confirmed that host-tissue injury is associated with NKG2D-mediated innate-like cytotoxicity of bystander memory CD8^+^ T cells activated by IL-15 during HAV infection (Kim et al., 2018). Notably, MHC class I-related chain A/B (MICA/B), the major ligand of NKG2D, was upregulated on virus-infected cells during hepatitis B virus (HBV) or other virus infections (Champsaur and Lanier, 2010). This led to the enhanced cytotoxicity mediated by bystander-activated CD8^+^ T cells.

In this study, bystander-activated CD8^+^ T cells specific to HTNV-unrelated viruses could be detected in the peripheral blood of HFRS patients. The elevated plasma IL-15 levels correlated with the disease severity and the expression of NKG2D on CD8^+^ T cells. HTNV-infected endothelial cells were found to be the source of elevated plasma IL-15. After analyzing the activation status and differentiated phenotypes of CD122^+^NKG2D^+^ bystander-activated CD8^+^ T cells in HFRS patients, we noticed that the IL-15-induced cytotoxic effects of bystander-activated CD8^+^ T cells on HTNV-infected endothelial cells could be blocked by the anti-NKG2D antibody. Taken together, we provided a possible mechanism of bystander activation of CD8^+^ T cells which mediated the endothelium injury through an innate-like cytolytic activity during HTNV infection in HFRS patients.

## Materials and methods

### Sample collection

The HFRS patients were recruited from the Department of Infectious Diseases at Tangdu Hospital of the Fourth Military Medical University (Xi’an, China) or from the Xi’an Eighth Hospital (Xi’an, China) from November 2020 to January 2021. A total of 74 HFRS patients infected with HTNV with ages ranging from 10 to 72 years were enrolled in the study. Sixteen healthy volunteers were enrolled as normal control, showing anti-HTNV negative or no HTNV risk factors. The clinical diagnosis of HFRS was confirmed by serological detection of HTNV-specific immunoglobulin M (IgM) or IgG antibodies. According to the clinical observation, the illness usually goes through five sequential stages: febrile, hypotensive, oliguric, diuretic, and convalescent, which could be roughly divided into two phases including acute phase (febrile, hypotensive, and oliguric stages) and convalescent phase (diuretic and convalescent stages). Mild, moderate, severe, and critical types of HFRS patients were classified based on the diagnostic criterion from the Prevention and Treatment Strategy of HFRS promulgated by the Ministry of Health, People’s Republic of China (**Supplementary Table 1**). We classified the patients into mild/moderate and severe/critical groups. In this case, the number of patients with mild/moderate and severe/critical disease severity was 33 and 41, respectively. In addition, patients with viral hepatitis, hematological diseases, autoimmune diseases, cardiovascular diseases, or other kidney disease were excluded from this study.

Informed consent was obtained from each HFRS patient or their guardians under a protocol approved by the Institutional Review Board of the Tangdu Hospital, the Xi’an Eighth Hospital, and the Fourth Military Medical University. The research involving human materials was also approved by the Ethical Review Board of the University with the license number KY20183312–1, and the related information was used anonymously.

Peripheral blood samples at different stages of HFRS were collected from each patient during hospitalization according to the previously described protocol (Zhang et al., 2021). A total of 58 plasma samples from different clinical stages of HFRS patients were collected. Peripheral blood mononuclear cells (PBMCs) were isolated by a standard Ficoll-Hypaque density gradient centrifugation. HLA-A*02 PBMCs were selected by staining with fluorescein phycoerythrin-HLA-A*02 monoclonal antibody (BB7.2, BioLegend) and applied in the following assays. The viral load was valued in our lab according to the previously described protocol (Yi et al., 2013). Other clinical indicators of each patient were recorded.

### Cells and viruses

Human umbilical vein endothelial cells (HUVECs) were isolated from human umbilical cords as previously described (Zhang et al., 2015) and cultured in endothelial cell medium (ECM), containing 10% fetal bovine serum (FBS), 1% penicillin-streptomycin, and 1% endothelial cell growth supplement (ScienCell, US). The cells were maintained at 37°C humidified atmospheres of 5% CO_2_ within eight passages. The HTNV 76-118 strain was kindly provided by the Department of Microbiology, the Fourth Military Medical University.

### *In vitro* infection

HUVECs were cultured in 6-well plates (4.5×10^5^ cells/well) and treated with no stimulation, or HTNV infection, or HTNV infection with tumor necrosis factor-α (TNF-α) stimuli, respectively. For the infection, the HUVECs were exposed to HTNV at multiplicity of infection of 2 for 2 h at 37°C. These cells were then washed with phosphate buffered saline (PBS) and re-incubated with or without TNF-α (20 ng/ml) in ECM. After 48 h post treatment, the culture supernatant was collected and the cells were harvested.

### Flow cytometry

For surface staining, 1×10^6^ PBMCs were suspended in each tube with 100 μL staining buffer (5% FBS in PBS) and incubated with the antibodies specific to the following surface markers, CD3/CD8/CD122/NKG2D/CD38/HLA-DR/CD45RA/CCR7. HUVECs with or without stimulation, including infected with HTNV, or HTNV infecting with TNF-α, were harvested and then stained with the antibodies specific to MICA/MICB and CD215 (IL-15Rα). The information of all the antibodies was summarized in **Supplementary Table 2**. The cells were incubated for 30 min at 4°C in the dark, followed by washing and resuspending in the staining buffer, and conducted on an ACEA Novo Express system (Agilent Bio).

For peptide/HLA-A*0201 tetramer staining, PE-labeled HLA-A*0201 tetramers refolded separately with the Epstein-Barr virus (EBV) nonapeptides (CLGGLLTMV) and cytomegalovirus (CMV) nonapeptides (NLVPMVATV) were customized by Epigen Biotech (Nantong, China). 3×10^6^ PBMCs of the patients were stained with each PE-labeled HLA-A*0201 tetramer for 10 min at room temperature, and subsequently stained with antibodies specific to CD3 and CD8 for 20 min at 4°C. Then the CD3^+^CD8^+^tetramer^+^ T cells were gated according to no-tetramer-stained isotype control (MOPC-21, BioLegend).

For intracellular cytokine staining, 3×10^6^ PBMCs from each HFRS patient were pre-treated with 20 μg/ml phorbol myristate acetate (PMA, eBiosciences), 1.5 μM ionomycin (eBiosciences), and monensin (eBiosciences) at 37 °C for 4 h. The PBMCs were stained with antibodies and tetramers against the surface markers. Then the cells were permeabilized with an intracellular staining kit (eBioscience) and incubated with the antibodies specific to granzyme B and perforin for 30 min at 4°C. HUVECs without stimuli, or infected with HTNV, or HTNV along with TNF-α stimuli were permeabilized and stained with antibody specific to the IL-15. Cells were washed and then resuspended for flow cytometry. Data analysis was performed using Flow Jo software (TreeStar).

For *in vitro* IL-15 stimulation assay, 3×10^6^ PBMCs were cultured with RPMI 1640 containing 10% FBS in 6-well plates in the presence of IL-15 (20 ng/mL, PeproTech) for 12 h. Then the cells were harvested and examined for expression of CD122, NKG2D, CD38, HLA-DR, CD45RA, CCR7, perforin or granzyme B in HLA tetramer^+^CD8^+^ T cells or CD122^+^NKG2D^+^ HLA tetramer^+^CD8^+^ T cells by flow cytometry following the protocol above.

### Enzyme-linked immunosorbent assay

The levels of IL-15 in plasma samples of HFRS patients or in the culture supernatant of HUVECs infected with HTNV were detected using an ELISA kit (D1500, R&D, US) respectively according to manufacturer’s instructions. SpectraMax Absorbance Reader (Molecular Devices, US) was used to determine the absorbance at 450 nm.

### Western blot

Cell extracts were prepared following the protocol of RIPA (ZHHC, China). Subsequently, 20 μg of total protein from each sample was separated by stacking gel and 4%–20% SDS-PAGE separating gel with a Tris-glycine system and transferred onto nitrocellulose membranes (Millipore). After blocking with 5% non-fat milk at room temperature for 1 h, the membrane was incubated with antibodies against GAPDH or MICA/B (Cell Signaling Technology, US) overnight at 4 °C, respectively. The membrane was then washed with Tris-buffered saline with Tween-20 and incubated with horseradish peroxidase-conjugated anti-rabbit IgG antibody (DIYIBio, China) for 1 h at room temperature. The immunoreactive bands were developed with the enhanced chemiluminescence reagent (Mishu, China).

### Cytotoxicity assay and antibody blocking assay

CD8^+^ T cells were isolated from the PBMCs of each HFRS patient using a CD8 positive selection kit (Miltenyi Biotec) according to the manufacturer’s instructions. The CD8^+^ T cells were then washed with RPMI 1640 containing 10% FBS, and incubated with or without 20 ng/ml IL-15 for 12 h, considered effector cells. HTNV-infected HUVECs labeled with 0.25 mM CFSE (Invitrogen) were used as target cells (Ayano et al., 2015). The CD8^+^ T cells treated with or without IL-15 were subsequently co-cultured with HTNV-infected HUVECs at effector (E): target (T) ratios of 5:1, 10:1 or 40:1 respectively at 37°C in 96-well plates. After 6 h co-culture, the HUVECs were collected and labeled with 7-aminoactinomycin D (7-AAD, BD Biosciences) at 4 °C for 5 min. The CFSE^+^7-AAD^+^ cells, defining as dead cells, were analyzed by flow cytometry (Ayano et al., 2015).

For antibody blocking assay, the isolated CD8^+^ T cells were incubated with or without 20 ng/ml IL-15 for 12 h. Then the NKG2D blocking antibody (10 µg/ml, BioLegend) was added and incubated at 37 °C for 20 min. Subsequently, the blocked CD8^+^ T cells were used as effector cells in the cytotoxicity assay.

### Statistical analysis

The statistical analysis was performed using GraphPad Prism 8.0 software. Comparisons between or among different groups were performed using the Mann-Whitney *U* test or the one-way ANOVA test. The value was presented as the mean value ± standard error (SE). Correlation analysis was performed using the Spearman correlation test. p values less than 0.05 were considered statistically significant.

## Results

### CD8^+^ T cells were activated and expressed NKG2D during HFRS

**Figure 1:**
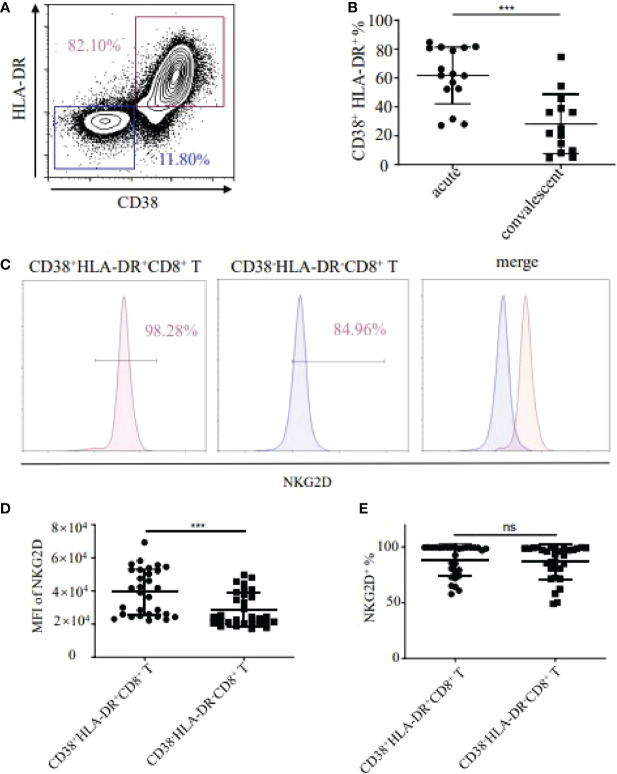
Detection of CD38^+^HLA-DR^+^CD8^+^ T cells and expression of NKG2D in HFRS patients. **(A)** Representative flow cytometry of CD38 and HLA-DR expression in CD8^+^ T cells. **(B)** Comparison of the frequencies of CD38^+^HLA-DR^+^ in CD8^+^ T cells between acute (n=16) and convalescent phase (n=14) in HFRS patients. **(C)** Representative flow cytometry of NKG2D expression in CD38^+^HLA-DR^+^ or CD38^-^HLA-DR^-^CD8^+^ T cells. **(D, E)** Comparison of the MFI **(D)** or the frequencies **(E)** of NKG2D between the CD38^+^HLA-DR^+^CD8^+^ T cells (n=30) and the CD38^-^HLA-DR^-^CD8^+^ T cells (n=30). The significance of the differences between two groups was determined by the Mann–Whitney *U*-test. p-Values<0.05 were considered to be statistically significant. ***p<0.001; “ns” means no significance.

Firstly, we examined the CD38^+^HLA-DR^+^ activation status of CD8^+^ T cells in the peripheral blood of HFRS patients. The results showed that the frequencies of CD38^+^HLA-DR^+^ activated CD8^+^ T cells in the acute phase of HFRS patients were higher than that in the convalescent stage (p<0.001, **Figures 1A, B**). We further detected the expression of NKG2D (Natural-killer group 2, member D) on CD38^+^HLA-DR^+^CD8^+^ T cells and CD38^-^HLA-DR^-^CD8^+^ T cells, respectively (**Figure 1C**). Although there was no significant difference in the frequencies of NKG2D^+^ cells between the two groups, the MFI (mean fluorescence intensity) of NKG2D on CD38^+^HLA-DR^+^CD8^+^ T cells was higher than that in CD38^-^HLA-DR^-^CD8^+^ T cells (p<0.001, **Figures 1D, E**). These results suggested that HTNV infection induced the activation of CD8^+^ T cells in HFRS patients, especially in the acute phase of HFRS. Moreover, the activated CD38^+^HLA-DR^+^CD8^+^ T cells expressed high levels of NKG2D molecule.

### Activated heterologous viral-specific CD8^+^ T cells expressed high levels of NKG2D in HFRS patients

**Figure 2:**
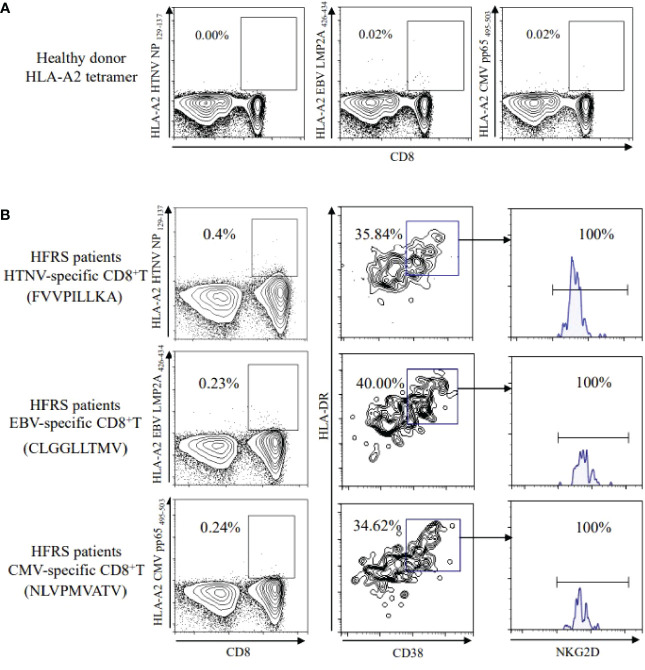
Detection of HTNV or other-virus specific CD8^+^ T cells in healthy donors and in HFRS patients. **(A)** HTNV, EBV, or CMV-specific CD8^+^ T cells in healthy donors. **(B)** Expression of NKG2D on CD38^+^HLA-DR^+^CD8^+^ T cells specific to HTNV, EBV, or CMV, respectively in HFRS patients.

To elucidate the antigen specificity of CD8^+^ T cells, the peptide/HLA-A*02 (p/HLA-A*02) tetramer^+^CD8^+^ T cells were detected. There were no HTNV epitope-specific CD8^+^ T cells and little frequencies of EBV or CMV epitope-specific CD8^+^ T cells detectable in healthy donors (**Figure 2A**). The frequencies of HTNV, EBV, CMV epitope-specific CD8^+^ T cells increased to 0.4%, 0.23%, and 0.24% in HFRS patients, respectively. In addition, the CD8^+^ T cells specific to HTNV, EBV, or CMV activated by showing CD38^+^HLA-DR^+^ phenotype. Importantly, the expression of cytolytic receptor NKG2D was detected on CD38^+^HLA-DR^+^ HTNV, EBV, or CMV-specific CD8^+^ T cells (**Figure 2B**). Thus, these results suggested that the memory CD8^+^ T cells specific to EBV or CMV were bystander activated and might exert function through NKG2D-dependent manner during HTNV infection.

### The elevated levels of plasma IL-15 in HFRS patients correlated with the disease severity

**Figure 3:**
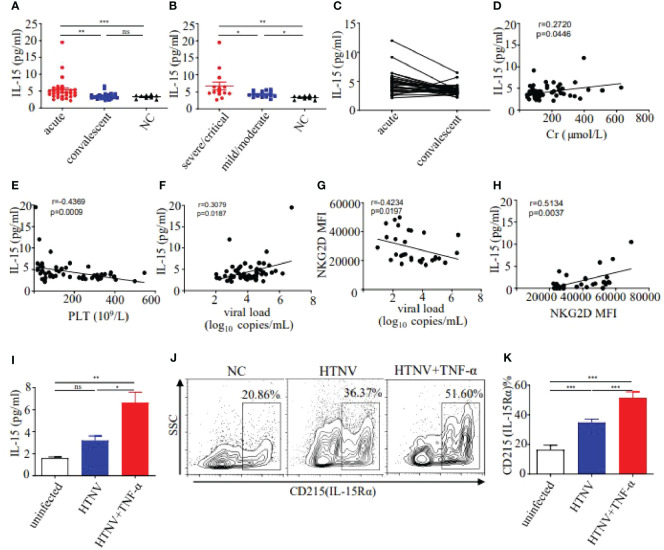
Detection of IL-15 levels in plasma and supernatant, and expression of IL-15Rα on HUVECs. **(A)** Comparison of the plasma IL-15 levels between acute (n=30) and convalescent phase (n=28) in HFRS patients. **(B)** Comparison of the plasma IL-15 levels in different severities of HFRS patients (both n=14). **(C)** The tendency in the plasma IL-15 levels from the acute to convalescent phases in HFRS patients (n=28). **(D, E)** Correlations between the plasma IL-15 levels and Cr levels (n=55) or PLT counts (n=55). **(F, G)** Correlations between the plasma HTNV RNA loads and plasma IL-15 levels (n=58) or NKG2D MFI (n=30). **(H)** Correlations between the plasma IL-15 levels and MFI of NKG2D on CD38^+^HLA-DR^+^CD8^+^ T cells in HFRS patients (n=30). **(I)** Comparison of the IL-15 levels in supernatant of HUVECs infected with HTNV (n=3), HTNV and TNF-α (n=3), or uninfected (n=3). **(J, K)** Representative flow cytometry **(J)** and comparison **(K)** of CD215 (IL-15Rα) expression on HUVECs infected with HTNV (n=4), HTNV and TNF-α(n=4), or uninfected(n=4), respectively. The significance of the differences between different groups was determined by the one-way ANOVA test. Spearman correlation test was used to evaluate the correlations. r denotes Spearman’s correlation coefficient. NC, normal control. *p<0.05; **p<0.01; ***p<0.001; “ns” means no significance.

Studies demonstrated that IL-15 was the critical cytokine inducing the bystander activation of CD8^+^ T cells (Kim et al., 2018). We further examined the levels of plasma IL-15 in HFRS patients. Results showed that the levels of plasma IL-15 in the acute phase of HFRS patients were higher than that in the healthy donors (5.256 ± 0.610 pg/ml vs. 3.492 ± 0.174 pg/ml, p<0.001) and were also higher than that in the convalescent phase of HFRS patients (3.258 ± 0.193 pg/ml, p<0.01, **Figure 3A**). Moreover, the levels of plasma IL-15 at the acute phase in severe/critical patients were significantly higher than that in mild/moderate patients (6.682 ± 1.178 pg/ml vs. 4.253 ± 0.243 pg/ml, p<0.05). The levels of plasma IL-15 at the acute phase in different severities of HFRS were higher than that in the healthy donors (p<0.01 and p<0.05, respectively) (**Figure 3B**). However, the plasma IL-15 levels only showed a declined tendency from acute to convalescent phases (**Figure 3C**). The correlation analysis revealed that the increasing levels of plasma IL-15 in HFRS patients were positively correlated with serum Cr (r=0.2720, p=0.0446, **Figure 3D**), while negatively correlated with the platelet counts (r=−0.4369, p=0.0009, **Figure 3E**) in HFRS patients, indicating the elevated plasma IL-15 might correlated with the HFRS disease severity. Moreover, the increasing viral load was positively correlated with plasma IL-15 levels (r=0.3079, p=0.0187, **Figure 3F**), while negatively correlated with NKG2D MFI on CD38^+^HLA-DR^+^CD8^+^ T cells (r=-0.4234, p=0.0197, **Figure 3G**). In addition, we found that the plasma IL-15 levels were positively correlated with the MFI of NKG2D on CD38^+^HLA-DR^+^CD8^+^ T cells in HFRS patients (r=0.5134, p=0.0037, **Figure 3H**). Thus, these results suggested that increasing IL-15 levels positively correlated with the disease severity during HTNV infection.

Considering that HTNV mainly infected endothelial cells in human, we used HUVECs (Human umbilical vein endothelial cells) infected with HTNV as an *in vitro* cell model to explore the source of the elevated IL-15 in HFRS patients. Results showed that IL-15 levels of cell supernatant in the HTNV-infected HUVECs group were 2-fold higher than that in the uninfected controls. It has been reported that the levels of proinflammatory cytokine TNF-α increased in HFRS patients (Kyriakidis and Papa, 2013), we further detected the level of IL-15 in HTNV-infected HUVECs combined with TNF-α stimulation. Notably, the IL-15 levels increased significantly in the HTNV-infected combined with TNF-α-stimulated group (6.634 ± 0.938 pg/ml) than that in the HTNV-infected alone group (3.213 ± 0.401 pg/ml, p<0.05) or than that in the control group (1.606 ± 0.101 pg/ml, p<0.01, **Figure 3I**). Thus, HTNV infection can induce the secretion of IL-15 from HUVECs, which is enhanced by the co-stimulation of TNF-α during HTNV infection.

Given that IL-15Rα could trans-present IL-15 to bind with IL-15Rβ to transmit the cell signal, we further examined the expression of IL-15Rα on HUVECs in different treatment groups. Results showed that the percentage of IL-15Rα on HUVECs in HTNV-infected group was higher than that in the uninfected group (34.86 ± 1.062% vs 16.55 ± 1.458%, p<0.001), while lower than that in HTNV-infected combined with TNF-α-stimulated group (51.54 ± 1.918%, p<0.001, **Figures 3J, K**), indicating that TNF-α have synergistic effects to induce the expression of IL-15Rα on HUVECs under the HTNV infection. These results suggested that HTNV-infected endothelial cells produce IL-15, which was correlated with the disease severity in HFRS patients.

### IL-15 treatment increased and activated the CD122^+^NKG2D^+^ bystander-activated CD8^+^ T cells in mild/moderate HFRS patients

**Figure 4:**
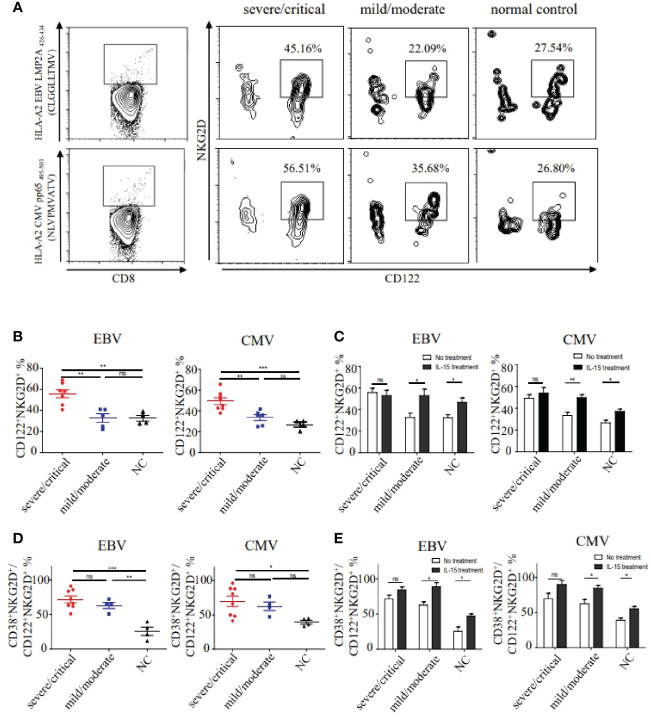
The frequencies and activation status of CD122^+^NKG2D^+^ EBV or CMV p/HLA-A*02 tetramer^+^CD8^+^ T cells in different severities of HFRS. **(A)** Representative flow cytometry of CD122^+^NKG2D^+^ phenotypes on EBV or CMV p/HLA-A*02 tetramer^+^CD8^+^ T cells in the severe/critical and mild/moderate patients. **(B)** Comparison of the frequencies of CD122^+^NKG2D^+^ EBV (n _severe/critical_ =7, n _mild/moderate_ =5) or CMV (n _severe/critical_ =8, n _mild/moderate_ =6) p/HLA-A*02 tetramer^+^CD8^+^ T cells between different severities of HFRS. **(C)** Comparison of the frequencies of CD122^+^NKG2D^+^ EBV or CMV p/HLA-A*02 tetramer^+^CD8^+^ T cells in severe/critical and mild/moderate of HFRS with or without IL-15 treatment. **(D)** Comparison of the frequencies of CD38^+^HLA-DR^+^CD122^+^NKG2D^+^ EBV (n _severe/critical_ =8, n _mild/moderate_ =4) or CMV (n _severe/critical_ =8, n _mild/moderate_ =4) p/HLA-A*02 tetramer^+^CD8^+^ T cells in the severe/critical and mild/moderate of HFRS. **(E)** Comparison of the frequencies of CD38^+^HLA-DR^+^CD122^+^NKG2D^+^ EBV or CMV p/HLA-A*02 tetramer^+^CD8^+^ T cells of HFRS with or without IL-15 treatment. *p<0.05; **p<0.01; ***p<0.001; “ns” means no significance.

Considering the correlation of IL-15 levels and NKG2D expression, we then examined the CD122 (IL-15Rβ) ^+^NKG2D^+^ phenotypes in EBV and CMV-derived bystander memory CD8^+^ T cells in PBMC (Peripheral blood mononuclear cell) of HFRS patients (**Figure 4A**). The frequencies of CD122^+^NKG2D^+^ EBV or CMV p/HLA-A*02 tetramer^+^CD8^+^ T cells in severe/critical patients were markedly higher than that in the mild/moderate HFRS patients (p _EBV_<0.01, p _CMV <_0.01), and were also higher than that in healthy donors (p _EBV_<0.01, p _CMV_<0.001, **Figure 4B**). Moreover, we studied the effects of IL-15 treatment on the expression of CD122 and NKG2D on bystander-activated CD8^+^ T cells in the absence of antigen stimulation *ex vivo*. As shown in **Figure 4C**, IL-15 stimulation significantly increased the frequencies of CD122^+^NKG2D^+^ EBV or CMV p/HLA-A*02 tetramer^+^CD8^+^ T cells in mild/moderate patients (p _EBV_<0.05, p _CMV_<0.01) and in the healthy donors (p _EBV_<0.05, p _CMV_<0.05).

We next analyzed the activation status of CD122^+^NKG2D^+^ EBV or CMV p/HLA-A*02 tetramer^+^CD8^+^ T cells in HFRS patients. Results showed that the frequencies of CD38^+^HLA-DR^+^CD122^+^NKG2D^+^ bystander-activated CD8^+^ T cells in the severe/critical patients were higher than that in the healthy donors (p _EBV_<0.001, p _CMV_<0.05, **Figure 4D**). Interestingly, compared to the healthy donors, the frequencies of CD122^+^NKG2D^+^ EBV p/HLA-A*02 tetramer^+^CD8^+^ T cells were significantly higher in the mild/moderate patients (p<0.01, **Figure 4D**). We then explored the effects of IL-15 treatment on the activation of CD122^+^NKG2D^+^ bystander CD8^+^ T cells. The results showed that the significant differences of the frequencies of CD122^+^NKG2D^+^ EBV or CMV p/HLA-A*02 tetramer^+^CD8^+^ T cells were observed only in mild/moderate patients and healthy donors after IL-15 stimulation (**Figure 4E**).

### IL-15 treatment induced effector memory-expressing CD45RA phenotype of CD122^+^NKG2D^+^ EBV or CMV p/HLA-A*02 tetramer^+^CD8^+^ T cells in mild/moderate HFRS patients

**Figure 5:**
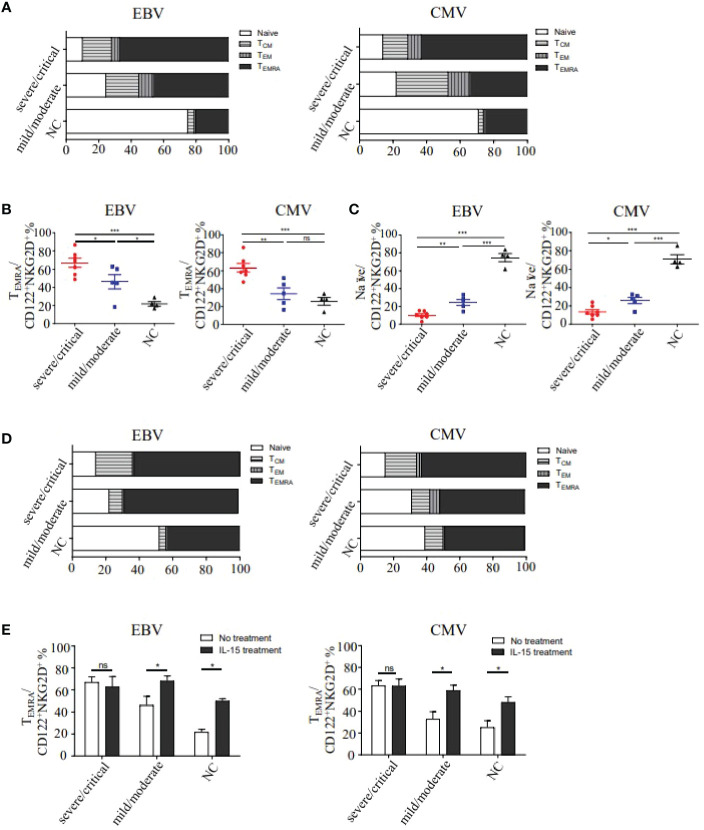
Analysis of the differentiation phenotypes of CD122^+^NKG2D^+^ EBV or CMV p/HLA-A*02 tetramer^+^CD8^+^ T cells in HFRS patients. **(A)** The memory phenotype constitution percentage of naïve (CD45RA^+^CCR7^+^), effector memory (CD45RA^-^CCR7^-^), central memory (CD45RA^-^CCR7^+^) and effector memory-expressing CD45RA (CD45RA^+^CCR7^-^) of CD122^+^NKG2D^+^ EBV or CMV p/HLA-A*02 tetramer^+^CD8^+^ T cells in HFRS. **(B)** Comparison of T_EMRA_ phenotype percentage in CD122^+^NKG2D^+^ EBV or CMV p/HLA-A*02 tetramer^+^CD8^+^ T cells between different severities of HFRS (n _EBV_ =12, n _CMV_ =12). **(C)** Comparison of Naïve phenotype percentage in CD122^+^NKG2D^+^ EBV or CMV p/HLA-A*02 tetramer^+^CD8^+^ T cells between different severities of HFRS (n _EBV_ =12, n _CMV_ =12). **(D)** The four memory phenotype constitution percentages of CD122^+^NKG2D^+^ EBV or CMV p/HLA-A*02 tetramer^+^CD8^+^ T cells in HFRS after IL-15 treatment (n _EBV_ =7, n _CMV_ =9). **(E)** Comparison of the T_EMRA_ percentage of CD122^+^NKG2D^+^ EBV or CMV p/HLA-A*02 tetramer^+^CD8^+^ T cells in different severities of HFRS with or without IL-15 treatment. *p<0.05; **p<0.01; ***p<0.001; “ns” means no significance.

To further analyze the effect phenotype of CD122^+^NKG2D^+^ bystander-activated CD8^+^ T cells, the expression of CD45RA and CCR7 were evaluated in HFRS patients. The results showed that effector memory-expressing CD45RA T cells (T_EMRA_) (CD45RA^+^CCR7^−^) was the dominant phenotype observed in CD122^+^NKG2D^+^ bystander-activated CD8^+^ T cells in severe/critical patients (median 59% _CMV_ and 62% _EBV_) and in mild/moderate (median 40% _CMV_ and 43% _EBV_), while the naïve phenotype (CD45RA^+^CCR7^+^) was the major phenotype in CD122^+^NKG2D^+^ bystander-activated CD8^+^ T cells in healthy donors (median 71% _CMV_ and 76% _EBV_, **Figure 5A**). As shown in **Figure 5B**, the frequencies of T_EMRA_ cells in CD122^+^NKG2D^+^ bystander-activated CD8^+^ T cells in severe/critical patients were significantly higher than that in mild/moderate patients (p _EBV_<0.05, p _CMV_<0.01), also higher than that in healthy donors (p _EBV_<0.001, p _CMV_<0.001). Similarly, the frequency of CD122^+^NKG2D^+^ EBV p/HLA-A*02 tetramer^+^CD8^+^ T cells of T_EMRA_ cells in the mild/moderate patients was significantly higher than that in healthy donors (p<0.05, **Figure 5B**). The frequencies of naïve cells in CD122^+^NKG2D^+^ bystander-activated CD8^+^ T cells in healthy donors were markedly higher than that in the severe/critical patients (p _EBV_<0.001, p _CMV_<0.001), also higher than that in the mild/moderate patients (p _EBV_<0.001, p _CMV_<0.001). The frequencies of naïve cell in the mild/moderate patients were higher than that in the severe/critical patients (p _EBV_<0.01, p _CMV_<0.05, **Figure 5C**).

We next explored the effects of IL-15 treatment on the phenotypes of CD122^+^NKG2D^+^ bystander-activated CD8^+^ T cells in the absence of antigen stimulation (**Figure 5D**). It remained no difference for the T_EMRA_ frequencies of CD122^+^NKG2D^+^ bystander-activated CD8^+^ T cells in severe/critical patients with or without IL-15 treatment. However, IL-15 treatment could increase the T_EMRA_ frequencies of CD122^+^NKG2D^+^ bystander-activated CD8^+^ T cells in mild/moderate patients (p _EBV_<0.05, p _CMV_<0.05) and in healthy donors (p _EBV_<0.05, p _CMV_<0.05, **Figure 5E**). Thus, IL-15 treatment induced the effectual function of CD122^+^NKG2D^+^ bystander-activated CD8^+^ T cells in mild/moderate patients.

### The bystander-activated CD8^+^ T cells from HFRS patients could exert NKG2D-dependent innate-like cytotoxic activity by IL-15 induction

**Figure 6:**
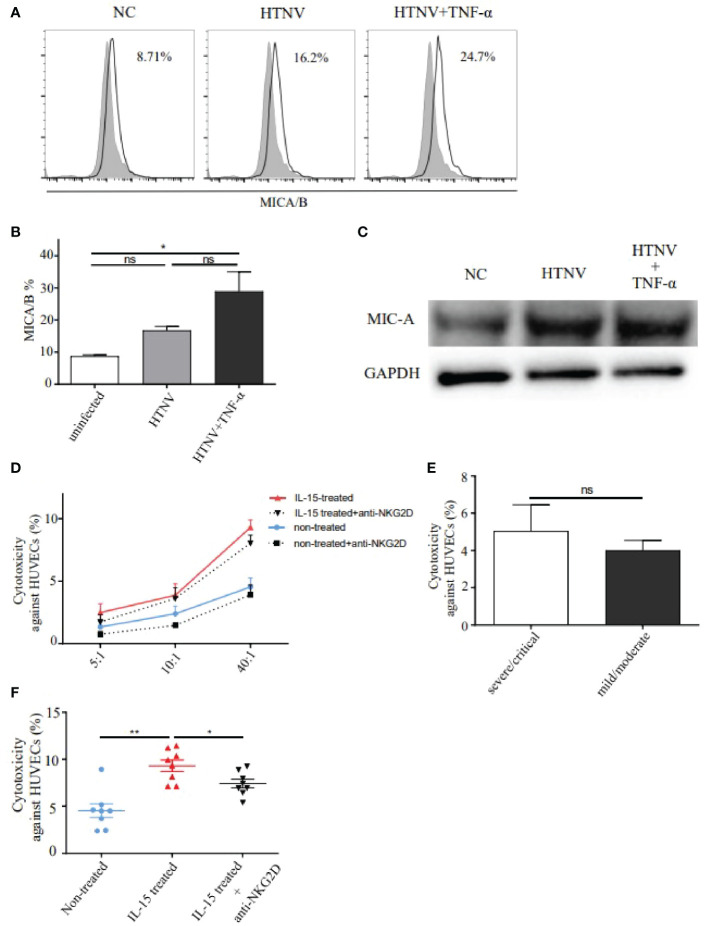
NKG2D mediated the cytotoxicity of IL-15-induced CD8^+^ T cells from HFRS patients against HUVECs infected with HTNV. **(A, B)** Representative flow cytometry **(A)** and comparison **(B)** of MICA/B expression on HUVECs infected with HTNV (n=3), HTNV and TNF-α (n=3), or uninfected (control) (n=3). **(C)** Western blot of MIC-A protein expression on HUVECs in different treatments. **(D)** HTNV-infected HUVECs were co-cultured with peripheral blood isolated CD8^+^ T cells (non-treated group, n=8), with CD8^+^ T cells combined with NKG2D neutralization (anti-NKG2D-blocked group, n=8), with CD8^+^ T cells induced by IL-15 (IL-15-treated group, n=8), and with CD8^+^ T cells induced by IL-15 and NKG2D neutralization (IL-15-treated with anti-NKG2D-blocked group, n=8) at **(E)** T ratios of 5:1, 10:1, and 40:1, respectively. **(E)** Comparison of the cytotoxicity of CD8^+^ T cells against HUVECs between the severe/critical and mild/moderate HFRS patients (both n=4) at ratios of 40:1. **(F)** Comparison of the cytotoxicity of CD8^+^ T cells against HUVECs among non-treated group (n=8), IL-15-treated group (n=8), and IL-15-treated with anti-NKG2D-blocked group (n=8). *p<0.05; **p<0.01; “ns” means no significance.

It was reported that NKG2D could bind with MICA/B and transmit cytolytic signals to the target cells. To confirm it, we further investigated the expression of MICA/B (MHC class I-related chain A/B) on HUVECs (**Figure 6A**). Results showed that the percentage of MICA/B on HUVECs in the HTNV-infected group was 1.9-fold higher than that in the control group. Similarly, the percentage of MICA/B on HUVECs increased significantly in the HTNV-infected combined with TNF-α-stimulated group compared with that in the control group (28.93 ± 6.122% vs. 8.71 ± 0.468%, p<0.05). There was no difference in MICA/B expression between the HTNV-infected alone group and the HTNV-infected combined with TNF-α-stimulated group (**Figure 6B**). Similar results of MIC-A protein expression increased in both the HTNV-infected group and HTNV-infected combined with TNF-α-stimulated group were further confirmed by western blot (**Figure 6C**).

The CD8^+^ T cell-mediated cytotoxicity assay was then performed to assess the effects of bystander-activated CD8^+^ T cells in HFRS patients. Since vascular endothelial cell injury has been considered one of the earliest events of HFRS, we selected HTNV-infected HUVECs as the target cells for CD8^+^ T cell-mediated cytotoxicity assay. Results showed that the percentages of cytotoxicity of CD8^+^ T cells in peripheral blood of HFRS against HUVECs were increased along with E: T (Effector: target) ratios of 5:1, 10:1, and 40:1 (**Figure 6D**). There was no significant difference in the percentage of cytotoxicity of CD8^+^ T cells against HUVECs between the severe/critical patients and the mild/moderate patients at an E: T ratio of 40:1 (**Figure 6E**). To explore whether NKG2D was involved in the cytotoxicity effects of bystander-activated CD8^+^ T cells, the cytotoxicity of CD8^+^ T cells against HTNV-infected HUVECs was carried out in the presence of anti-NKG2D blocking antibody. Notably, the percentage of cytotoxicity was significantly decreased at each E: T ratio in the anti-NKG2D-blocked group (**Figure 6D**).

The effects of IL-15 treatment on the cytolytic activity of CD8^+^ T cells were further detected in the cytotoxic assay. Results showed that there was a higher percentage of cytotoxicity of CD8^+^ T cells against HUVECs in the IL-15-treated group than that in the non-treated group at an E: T ratio of 40:1 (p<0.01, **Figure 6F**). Similarly, the percentage of cytotoxicity of CD8^+^ T cells against HUVECs could be significantly inhibited by NKG2D-blocked antibody at each ratio, which was still higher than that in the non-treated group (**Figure 6D**). The results showed that significant difference in cytotoxicity against HUVECs at the ratio of 40:1 between the IL-15-treated group and IL-15-treated with anti-NKG2D-blocked group (p<0.05, **Figure 6F**). Thus, the above data suggested that bystander-activated CD8^+^ T cells from HFRS patients exerted NKG2D-dependent cytotoxic activity by IL-15 and mediated the injury of HTNV-infected HUVECs.

## Discussion

The effects of bystander activation of memory CD8^+^ T cells were controversial among many human viral infections (Kim and Shin, 2019; Lee et al., 2022). Here, we proved for the first time that there was bystander activation of EBV or CMV-specific memory CD8^+^ T cells during HTNV infection in HFRS patients. HTNV-infected endothelial cells secreted IL-15, which could induce CD122^+^NKG2D^+^ EBV or CMV p/HLA-A*02 tetramer^+^CD8^+^ T cells response with high frequencies, excessive activation status and effector memory-expressing CD45RA phenotypes in HFRS patients. In addition, CD122^+^NKG2D^+^ bystander-activated CD8^+^ T cells showed strong cytotoxicity. Importantly, the expression of NKG2D ligand-MICA/B elevated on HTNV-infected endothelial cells, which potentially performed the targets for innate-like cytolytic bystander-activated CD8^+^ T cells. We provided a possible mechanism of CD8^+^ T cells bystander activation mediated by the IL-15/NKG2D axis, which may be helpful to reveal the role of bystander-activated CD8^+^ T cells for the immunopathological injury during viral infection.

Our previous studies suggested that HTNV-specific IFN-γ-producing CD8^+^ T cells primarily contributed to the protection against severe acute renal failure caused by HTNV infection in HFRS patients (Wang et al., 2009). Differently, we focused innovatively on the HTNV-unrelated memory CD8^+^ T cells in this study, which were bystander-activated and exerted innate-like cytotoxicity leading to pathological injury of endothelial cells in HFRS patients. Indeed, bystander-activated CD8^+^ T cells have been proven to be associated with tissue injury during many viral infections in humans, such as liver injury in acute hepatitis A patients (Kim et al., 2018) and in HBV patients (Maini et al., 2000). Moreover, the study on human immunodeficiency virus infection suggested that the expansion and granzyme B secretion of bystander-activated CD8^+^ T cells were associated with increased morbidity and mortality in patients (Younes et al., 2016). Conflicting results of immunological roles of CD8^+^ T cells during HTNV infection may be due to the activation through different pathways. HTNV-specific CD8^+^ T cells were activated through TCR-mediated pathway by binding to cognate peptide presented by major histocompatibility complex class I. But HTNV-unrelated CD8^+^ T cells specific to such as EBV or CMV were activated without corresponding cognate peptide during HFRS. The bystander-activated CD8^+^ T cells did not recognize the infected cells through TCR, resulting innate-like cytotoxicity of the bystander-activated CD8^+^ T cells.

It has been demonstrated that EBV and CMV-specific CD8^+^ T cells could be bystander-activated in patients with HAV, HBV, or dengue virus (DENV) infections (Sandalova et al., 2010; Kim et al., 2018). Consistently, we found that EBV or CMV-specific CD8^+^ T cells could express high levels of CD38^+^HLA-DR^+^ activated phenotypes in HFRS patients but not in healthy donors. On one hand, EBV and CMV are the most ubiquitous herpes viruses, with a prevalence of up to 95% for EBV and close to 50% for CMV in the adult population globally (Muller-Durovic et al., 2019). Following primary infection, both EBV and CMV could establish life-long latent infection (Muller-Durovic et al., 2019). On the other hand, memory CD8^+^ T cells are more sensitive to cytokine-mediated signals than antigen-inexperienced T cells and can be triggered to release their cytotoxic contents when exposed to far lower cytokine concentrations than naïve T cells (Whiteside et al., 2018). Indeed, not only for EBV and CMV-specific CD8^+^ T cells, the influenza A virus, respiratory syncytial, or vaccinia virus-specific CD8^+^ T cells also could be bystander activated during HAV infection (Kim et al., 2018). As diversities of viral infections in different individuals, we believed that besides CMV and EBV, there may be other viruses which could also induce bystander-activated CD8^+^ T cells during HTNV infection, holding an attractive part of total CD8^+^ T cells in HFRS patients.

The levels of IL-15 in peripheral blood or tissue have been shown to be increased in many viral infections, such as HIV (Leeansyah et al., 2013; Younes et al., 2016), HAV (Kim et al., 2018), simian immunodeficiency virus (Jacquelin et al., 2014), DENV (Azeredo et al., 2006), and respiratory syncytial virus infection (Estripeaut et al., 2008). Specifically, the HAV-infected hepatocytes were considered to be the main source of IL-15. Moreover, the levels of IL-15 were associated with bystander activation of CD8^+^ T cells in the case of acute hepatitis A patients. In this study, we found that HUVECs infected with HTNV or stimulated synergistically with TNF-α could be one of the major sources of elevated IL-15. Further studies are still needed to find whether other lymphocytes or tissue cells would be involved in the production of IL-15 during HTNV infection. However, how the IL-15 exerted its function is unclear. It has been reported that IL-15-treated CD8^+^ T cells could overexpress NKG2D and NKp30 and exert innate-like cytotoxicity in an NKG2D and NKp30-dependent manner (Kim et al., 2018). It has also been demonstrated that endogenous DNAX-activating protein 10 (DAP10), one of the important signaling adaptors transmitting signals for NKG2D (Lanier, 2005), bound specifically to the IL-15Rβ (CD122)-γ-chain (CD132) on cells (Horng et al., 2007). Moreover, the NKG2D-IL-15 signaling pathway has been found to be involved in CD8^+^ T-cell-mediated progressive muscle destruction in inflammatory myopathies (Ruck et al., 2015). Although our current study revealed that elevated plasma IL-15 levels were correlated with the HFRS severity and were associated with the expression of NKG2D on bystander-activated CD8^+^ T cells, a further study focusing on the downstream molecular pathway of the IL-15/NKG2D axis will be needed.

Granzyme B and perforin were important cytotoxic mediators of cytotoxic lymphocytes. In this study, our results suggested that CD122^+^NKG2D^+^ bystander-activated CD8^+^ T cells could produce abundant granzyme B and perforin (**Supplementary Figures 1**, **2**). Indeed, NKG2D can directly bind to a diverse family of ligands on the surface of target cells, thereby resulting in the activation or synergistic stimulation of immune effectors and subsequently release of cytolytic molecules (Jones et al., 2022). Thus, NKG2D^+^ cells might be positive totally for the expression of granzyme and perforin. In addition, we supposed that the high levels of IL-15 in severe/critical patients might be a strong stimulation for the CD122^+^NKG2D^+^ bystander-activated CD8^+^ T cells to a restricted degree *in vivo*, resulting that extra IL-15 treatment *in vitro* could not further induce the increased frequencies and phenotypes of bystander-activated CD8^+^ T cells. Consistently, the frequencies and phenotypes of CD122^+^NKG2D^+^ bystander-activated CD8^+^ T cells after IL-15 stimulation in mild/moderate HFRS patients or in healthy donors were remain lower than that in the severe/critical HFRS.

The immunopathological role of bystander-activated CD8^+^ T cells has been reported in many viral-infected diseases (Kim et al., 2018; Kim and Shin, 2019; Lee et al., 2022). The IL-15/NKG2D axis, serving as an important pathway of the bystander activation of memory CD8^+^ T cells during HTNV infection, was found in this study as a possible mechanism for the pathogenesis of HFRS. However, the study still has some limitations. The relationship between HTNV replication and IL-15 levels were still not be demonstrated *in vitro*. The underlying downstream molecular of the IL-15/NKG2D axis in bystander activation of CD8^+^ T cells was unraveled during HTNV infection. Our current study further raises the question that whether the IL-15/NKG2D axis would contribute to bystander activation of CD8^+^ T cells in other human viral diseases, and whether IL-15 or NKG2D could be considered as a therapeutic target in the treatment of the immunopathology of viral diseases.

## Data availability statement

The original contributions presented in the study are included in the article/**Supplementary Material**, further inquiries can be directed to the corresponding author/s.

## Ethics statement

The studies involving human participants were reviewed and approved by Ethical Review Board of the University with the license number KY20183312-1. Written informed consent to participate in this study was provided by the participants’ legal guardian/next of kin.

## Author contributions

YM and YZ conceptualized the study and designed the experiments. XZ, YSZ, and HL conducted the experiments. KT, CZ, MW, and MX helped with the literature research and analyzed the experimental data. XJ, HH, FZ, and NL collected the patient samples and analyzed the clinical data. RZ, BJ, YM, and YZ assisted with the manuscript revision and final approval for submission. All authors contributed to the article and approved the submitted version.
