# Supplementary figures and images for: IL-15 induced bystander activation of CD8+ T cells may mediate endothelium injury through NKG2D in Hantaan virus infection
Source: Front Cell Infect Microbiol. 2022 Dec 15;12:1084841. doi: 10.3389/fcimb.2022.1084841 (PMC9797980; doi:10.3389/fcimb.2022.1084841)

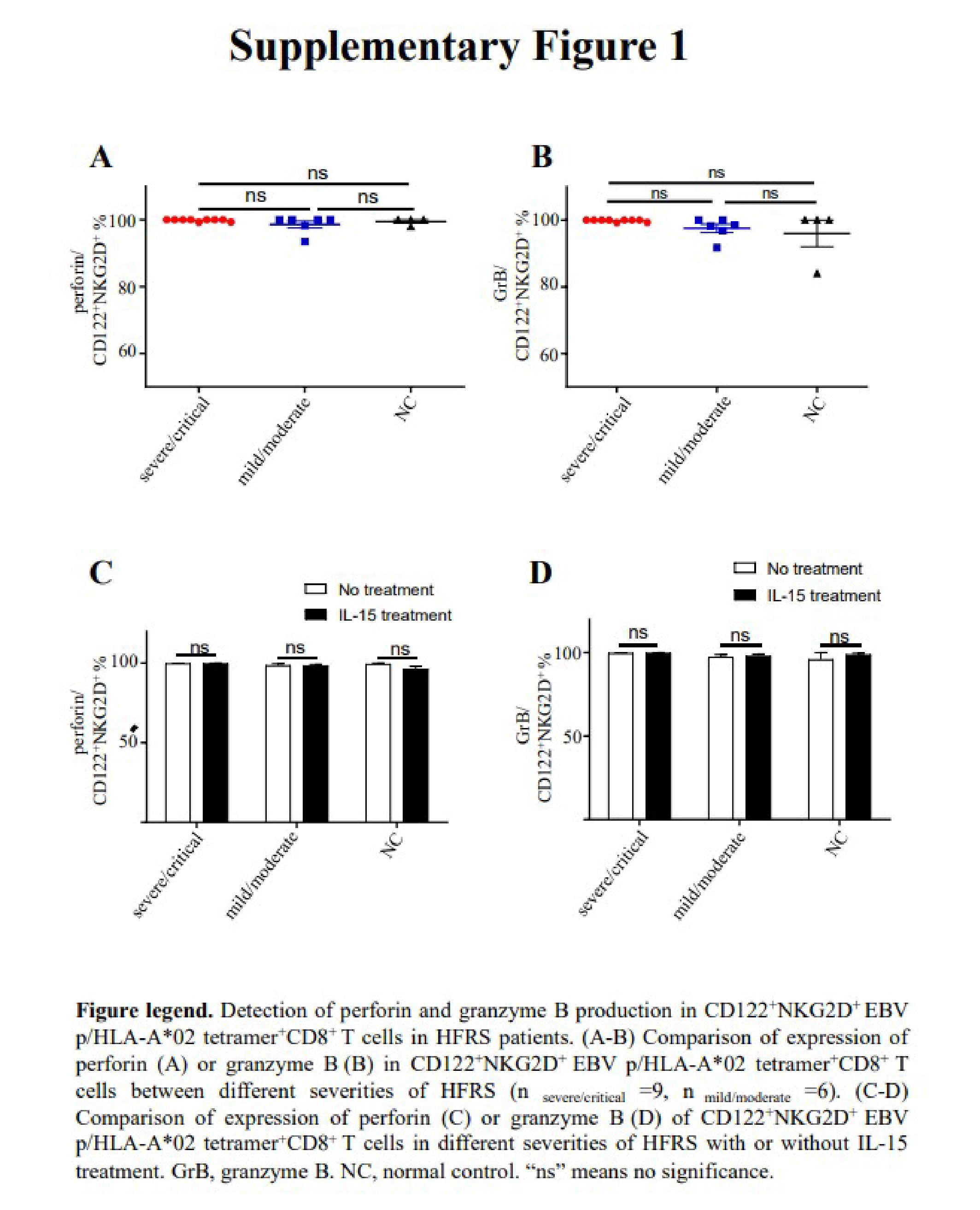

Supplement: Supplementary file 1 [file Image_1.jpeg]

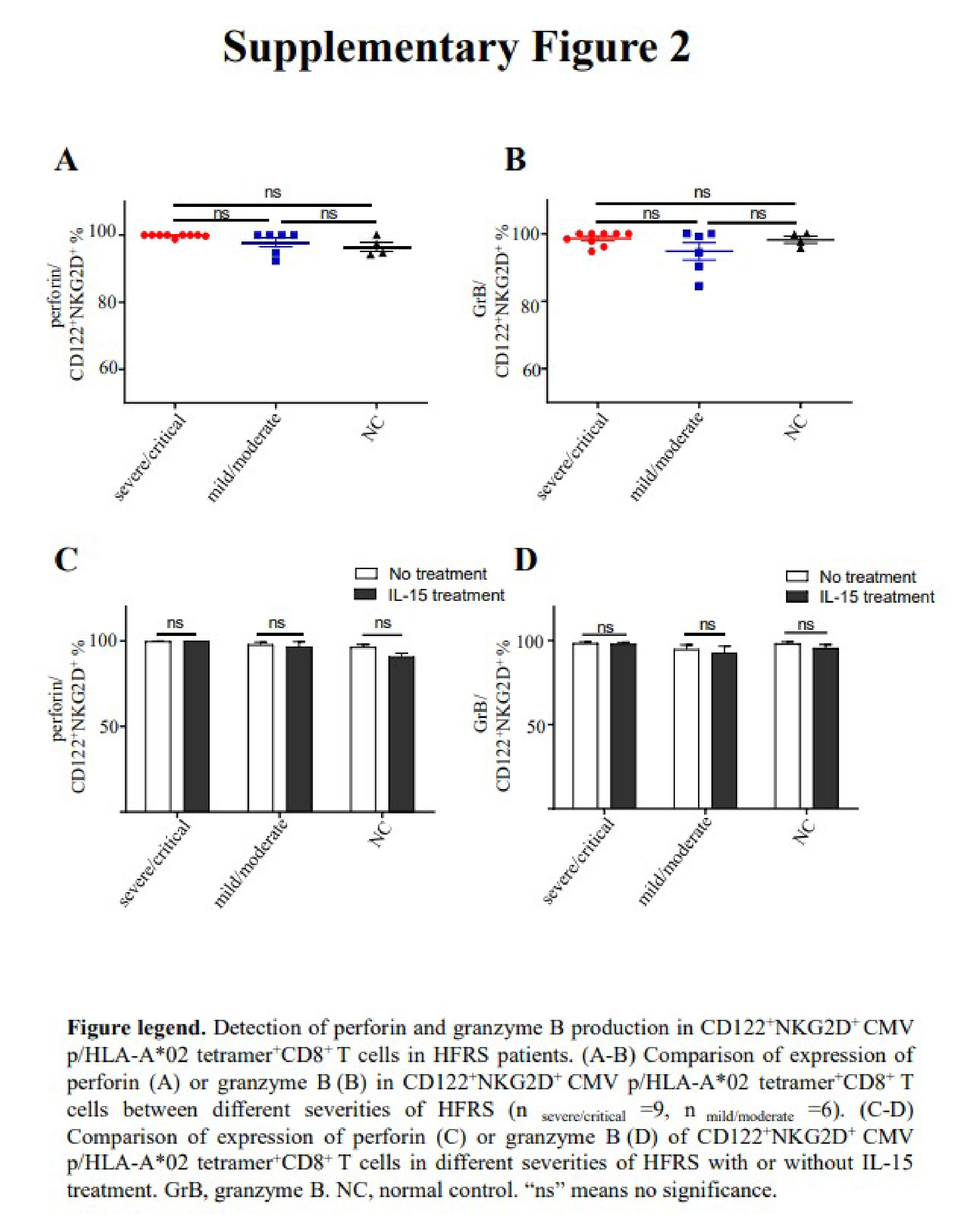

Supplement: Supplementary file 2 [file Image_2.jpeg]
