# Supplementary material for: IL-15 induced bystander activation of CD8+ T cells may mediate endothelium injury through NKG2D in Hantaan virus infection
Source: Front Cell Infect Microbiol. 2022 Dec 15;12:1084841. doi: 10.3389/fcimb.2022.1084841 (PMC9797980; doi:10.3389/fcimb.2022.1084841)
Supplement: Supplementary file 3 [file Table_1.docx]

Supplementary table 1. Four clinical types of HFRS patients

| HFRS types | Clinical symptom |
| --- | --- |
| mild | mild renal failure without an obvious oliguric stage |
| moderate | obvious symptoms of uremia, effusion (bulbar conjunctiva), hemorrhage (skin and mucous membrane), renal failure with a typical oliguric stage |
| severe | severe uremia, effusion (bulbar conjunctiva and either peritoneum or pleura), hemorrhage (skin and mucous membrane), and renal failure with oliguria (urine output, 50-500 mL/day) for ≤5 days or anuria (urine output, <50 mL/day) for ≤2 days |
| critical | for those with ≥1 of the following symptoms: refractory shock, visceral hemorrhage, heart failure, pulmonary edema, brain edema, severe secondary infection, and severe renal failure with oliguria (urine output, 50–500 mL/day) for >5 days, anuria (urine output, <50 mL/day) for >2 days, or a blood urea nitrogen (BUN) level of >42.84 mM |
