# Supplementary material for: IL-15 induced bystander activation of CD8+ T cells may mediate endothelium injury through NKG2D in Hantaan virus infection
Source: Front Cell Infect Microbiol. 2022 Dec 15;12:1084841. doi: 10.3389/fcimb.2022.1084841 (PMC9797980; doi:10.3389/fcimb.2022.1084841)
Supplement: Supplementary file 4 [file Table_2.docx]

| **antibody** | **fluorescent** | **Clone No.** | **company** |
| --- | --- | --- | --- |
| CD3 | BV-605 | OKT3 | Biolegend |
| CD8 | PerCP/Cy5.5 | SK1 | Biolegend |
| CD122 | APC | TU27 | Biolegend |
| NKG2D | PE/Cy7 | 1D11 | Biolegend |
| CD38 | BV421 | HTT2 | Biolegend |
| HLA-DR | FITC | L243 | Biolegend |
| CD45RA | FITC | HI100 | Biolegend |
| CCR7 | BV421 | G043H7 | Biolegend |
| MICA/B(FCM) | Alexa Fluor 488 | 6D4 | Biolegend |
| CD215 | PE | JM7A4 | Biolegend |
| Granzyme B | FITC | QA16A02 | Biolegend |
| Perforin | BV421 | dG9 | Biolegend |
| IL-15  GAPDH | APC  Protein | APC  5174S | Invitrogen  Cell Signaling Technology |
| MICA/B | Protein | 64899S | Cell Signaling Technology |
| NKG2D blocking | Blocking antibody | CD314 | Biolegend |

Supplementary Table 2: Information of antibodies used in the study.
